# The Usefulness of qPCR Data for Sample Pre-Assessment and Interpretation of Genetic Typing Results

**DOI:** 10.3390/genes15060744

**Published:** 2024-06-05

**Authors:** Martina Onofri, Simona Severini, Federica Tommolini, Massimo Lancia, Cristiana Gambelunghe, Luigi Carlini, Eugenia Carnevali

**Affiliations:** 1 Section of Legal Medicine, Department of Medicine and Surgery, Santa Maria Hospital, University of Perugia, 05100 Terni, Italy; s.severini@aospterni.it (S.S.); f.tommolini@aospterni.it (F.T.); e.carnevali@aospterni.it (E.C.); 2 Section of Legal Medicine, Department of Medicine and Surgery, University of Perugia, 06123 Perugia, Italy; massimo.lancia@unipg.it (M.L.); cristiana.gambelunghe@unipg.it (C.G.); luigi.carlini@unipg.it (L.C.)

**Keywords:** human DNA quantification, qPCR variation, forensic genetics, probative STR profile, average peak height, trace samples, casework samples

## Abstract

DNA quantification is a crucial step in the STR typing workflow for human identification purposes. Given the reaction’s nature, qPCR assays may be subjected to the same stochastic effects of traditional PCR for low-input concentrations. The study aims to evaluate the precision of the PowerQuant^®^ (Promega) kit assay measurements and the degree of variability for DNA templates falling below the optimal threshold of the PowerPlex^®^ ESX-17 Fast STR typing kit (Promega). Five three-fold dilutions of the 2800 M control DNA (Promega) were set up. Each dilution (concentrations: 0.05, 0.0167, 0.0055, 0.00185, and 0.000617 ng/µL) was quantified and amplified in four replicates. Variability for qPCR results, STR profile completeness, and EPGs’ peak height were evaluated. The qPCR-estimated concentration of casework samples was correlated with profile completeness and peak intensity, to assess the predictive value of qPCR results for the successful STR typing of scarce samples. qPCR was subjected to stochastic effects, of which the degree was inversely proportional to the initial input template. Quantitation results and the STR profile’s characteristics were strongly correlated. Due to the intrinsic nature of real casework samples, a qPCR-derived DNA concentration threshold for correctly identifying probative STR profiles may be difficult to establish. Quantitation data may be useful in interpreting and corroborating STR typing results and for clearly illustrating them to the stakeholders.

## 1. Introduction

The detection and qualitative and quantitative characterization of biological evidence is a crucial step in the workflow of forensic genetics laboratories. Human DNA quantification is strongly recommended by international guidelines in accordance with the highest quality standards in human identification [1]. This quantitative analysis makes it possible to accurately estimate the amount of nuclear DNA present in a sample and to calculate the optimal input of DNA template for downstream Polymerase Chain Reaction (PCR) amplification, thus increasing the probability of retrieving high-quality, probative STR (Short Tandem Repeats) profiles [2]. Insufficient template DNA may result in lower peak height, leading to an increased probability of allele drop-out and a higher degree of heterozygous imbalance, while an excessive amount of starting material may cause off-scale peak height, split peaks, pull-up and stutter peaks, and off-scale peak heights [3,4,5,6].

For its sensitivity, specificity, accuracy, precision, and multiplexing ability, quantitative real-time PCR (qPCR) is the gold standard for human DNA quantification in current forensic practices [7,8,9,10,11,12]. qPCR is a PCR-based technique in which the presence of fluorescent reporter molecules allows for the real-time detection of PCR amplicons. A mathematical model correlates the emitted fluorescence to the amount of DNA copies generated during the PCR cycles, thus inferring the original template concentration. Several chemistries are available, and their rationale has been thoroughly described in the noteworthy review by [13]. Through the employment of a variety of probes, qPCR can provide exhaustive qualitative information on any given sample. In fact, successful STR typing is heavily dependent not only on the quantity of amplifiable nuclear DNA present in the extract but also on its degree of degradation and on the presence of PCR inhibitors, which may impair the analysis despite the presence of an adequate amount of starting material [14,15,16]. In addition to homemade assays, commercial qPCR kits are available for forensic use [10,11,17,18,19].

In capillary electrophoresis (CE) electropherograms (EPG), the signal intensity (RFU) for each detected allele is strongly correlated with the amount of DNA template used in the STR PCR amplification step (herein shortened as STR-PCR) [12,20,21,22,23]. Therefore, alternative approaches suggest the inference of DNA concentration in a sample from the mean peaks’ height [12]. However, the DNA quantitation step may often offer the opportunity for a pre-assessment of the samples for a cost and time-efficient optimization of the laboratory workflow [24]. Samples that may yield informative profiles are PCR amplified, while samples of insufficient quantity (according to the PCR kit’s requirements) may be excluded from further processing. However, given the rationale behind qPCR assays, it is expected that qPCR may be subjected to the same stochastic effects and inherent inefficiency that characterize traditional PCR for low-quality DNA templates, leading to analytical variability [25,26,27]. Additionally, due to the intrinsic nature of real casework samples, a qPCR-derived DNA concentration threshold for correctly identifying probative STR profiles may be difficult to establish [17,24,28,29]. Quantitation data may be useful in interpreting and corroborating STR typing results and serve the purpose of clearly illustrating them to the stakeholders.

Three were the main aims of this study:

(A) Measuring the precision of the qPCR assay for DNA templates of poor quantity but ideal quality and the degree of variability caused by non-systematic, stochastic effects.
(B) Assessing how well the qPCR-measured DNA template amount is correlated with the qualitative and quantitative characteristics of an STR profile, as well as with the variability observed in STR-PCR results.
(C) Evaluating the impact of DNA amount and other factors on the retrieval of an informative STR profile in real casework samples of varying concentration, degradation, and purity.

In this study, the PowerQuant^®^ System (Promega, Madison, WI, USA) has been employed. The sensitivity, accuracy, and precision of the PowerQuant^®^ System have already been described in the literature [10,24,29]. Our laboratory’s internal validation of the kit confirmed the results and led to the implementation of the PowerQuant^®^ System in our laboratory’s workflow for routine casework analysis. Thanks to four proprietary probes of undisclosed sequence [30], the PowerQuant^®^ System makes it possible to infer forensically relevant, quantitative and qualitative information on a sample. The total amount of amplifiable, human DNA in the sample is measured through the small, autosomal “Auto” target concentration. The integrity of the sample (Degradation Index, DI) is inferred from the ratio [Auto]/[Deg], where “Deg” is a longer autosomal amplicon. The greater the ratio between the two targets’ concentrations is, the more degraded the DNA, thus predicting a higher chance of allele drop-out for the higher molecular weight loci. PCR inhibitors are detected through an internal PCR control (IPC Shift), and lastly, two multicopy targets on the Y chromosome allow for increased sensitivity to the male component of a mixture.

In this study, qPCR and STR-PCR variation only caused by quantity-dependent stochastic effects, while removing the additional variables of degradation and inhibition, was assessed. Five three-fold serial dilutions of a control DNA line, with an “Auto” target concentration ([Auto]) falling at or below the optimal threshold for STR amplification, were quantified and amplified in replicates. For each dilution in the series, information was thus inferred on the amount of amplifiable DNA ([Auto]), the variability of the measurement across qPCR replicates, STR profiles’ completeness, and peak height variation across STR-PCR replicates. [Auto] data were correlated with profile completeness, mean peak height, and peak height variation. Additionally, quantitation data collected from scarce casework samples were correlated with the respective STR profiles’ completeness and peak intensity, to assess the predictive value of qPCR results for the successful STR typing of scarce samples characterized by varying degrees of degradation (DI) and purity (IPC Shift).

## 2. Materials and Methods

The study was approved by the Ethics Committee of the University of Perugia (prot. n. 114846, app. 25 March 2024). Data were collected from both control DNA samples and casework samples.

### 2.1. Control DNA Dilutions—Laboratory Protocol and Statistical Analysis

To gauge qPCR and STR-PCR variation only caused by quantity-dependent stochastic effects, while removing degradation and inhibition-caused variability, the 2800 M control DNA (cat.# DD7101, Promega, Madison, WI, USA) was used. The product was diluted with sterile water to an initial concentration of 0.05 ng/μL, and a series of three-fold dilutions was set up for a total of five samples with the following concentrations: 0.05, 0.0167, 0.0055, 0.00185, and 0.000617 ng/μL. The five dilutions were assigned a letter from A to E in descending order of concentration. Such low concentrations may often be encountered in forensic casework when collecting trace evidence. In particular, the five concentrations herein studied were chosen to be equal to or below the optimal DNA concentration for the PowerPlex^®^ ESX-17 Fast System (0.05 ng/μL) and to provide enough data points for inferring the correlation between the true concentration and the qualitative and quantitative characteristics of interest to the study, meaning qPCR-measured [Auto] concentration, STR profile completeness, and peak height.

The samples were quantified in four technical replicates with the PowerQuant^®^ System (Promega, Madison, WI, USA) [10,30] on the Applied Biosystems™ 7500 Real-Time PCR instrument (Thermo Fisher Scientific, Waltham, MA, USA). Four qPCR data points were obtained per dilution for 20 total determinations.

Each dilution was STR-PCR amplified neat four times with the PowerPlex^®^ ESX-17 Fast kit (Promega, Madison, WI, USA), as per manufacturer’s instructions [31,32], on the Mastercycler^®^ ep thermal cycler (Eppendorf, Hamburg, Germany). Fragment separation was carried out with CE instrument Applied Biosystems™ SeqStudio™ Genetic Analyzer (Thermo Fisher Scientific, Waltham, MA, USA). Run conditions were the following: injection time, 7 s; injection voltage, 1200 V; run time, 1440 s; run voltage, 9000 V. The resulting CE data was analysed with GeneMapper^®^ ID-X v 1.6 (Thermo Fisher Scientific, Waltham, MA, USA). The analytical threshold (AT) and the stochastic threshold (ST) were established after internal validation of the laboratory’s analysis protocol, per national and international guidelines [33,34,35], and were both set to 50 RFU. Each PCR replica was CE separated once, for a total of four EPGs per dilution, thus resulting in a total of 20 control DNA EPGs.

To minimize system variation, one laboratory staff member performed all wet laboratory steps, reducing pipetting error, in a single assay. Since working with highly concentrated commercial control DNA, a negative control was quantified and PCR-amplified together with the dilutions.

Precision and accuracy of the PowerQuant^®^ System were evaluated, and the impact of stochastic effects on qPCR measurements for small DNA concentration was assessed. Variation in quantification results was correlated with STR-PCR variation. For each dilution, qualitative and quantitative features of the EPGs, such as profile completeness, drop-ins, peak height, and peak height ratio, were analyzed in correlation to the estimated DNA concentration. Since drop-in alleles are stochastic STR-PCR events, they were not considered in the quantitative statistical analysis. STR profile completeness was calculated based on the consensus profile. Alleles were included in the consensus profile if they appeared in 3 out of 4 replicates (n/2 + 1 replicates, n being the total number of replicates [36]). For accurate peak height and derived measurements calculations, all alleles were included in the genotype, while missing values (drop-out alleles) were assigned a height = 0 RFU.

### 2.2. Casework Samples—Statistical Analysis

Qualitative and quantitative information from 60 casework samples, collected and analyzed over time by the Forensic Genetics Laboratory of Santa Maria Hospital in Terni, was selected. The samples showed a wide range of qualitative characteristics (e.g., IPC, DI); however, they all returned an [Auto] of less than 1 ng/μL. To minimize variation due to different extraction methods, samples extracted with the phenol-chloroform method were chosen [37]. All samples had been previously analyzed according to the internal laboratory’s protocol. qPCR, STR-PCR, CE run, and fragment analysis conditions were those reported in Section 2.1.

The data was completely anonymized, aggregated, and analyzed. qPCR-estimated concentration, EPGs peak intensity, drop-out loci, and suitability for comparison of the trace were evaluated. The number of STR-PCR replicates for each sample varied (2–4); therefore, mean EPG peak height and number of drop-out loci were calculated by grouping replicates. A trace was considered suitable for comparison if the consensus profile showed alleles in at least 10 loci, as defined by the guidelines for personal identification by the Italian Speaking Working Group (Ge.F.I., Genetisti Forensi Italiani) of the International Society for Forensic Genetics (ISFG) [35].

Data visualization, data wrangling, and statistical analysis were performed using R v. 4.2.2.

## 3. Results

### 3.1. 2800 M Control DNA Dilutions

#### 3.1.1. PowerQuant^®^ System accuracy, precision, and stochastic variation

**Figure 1:**
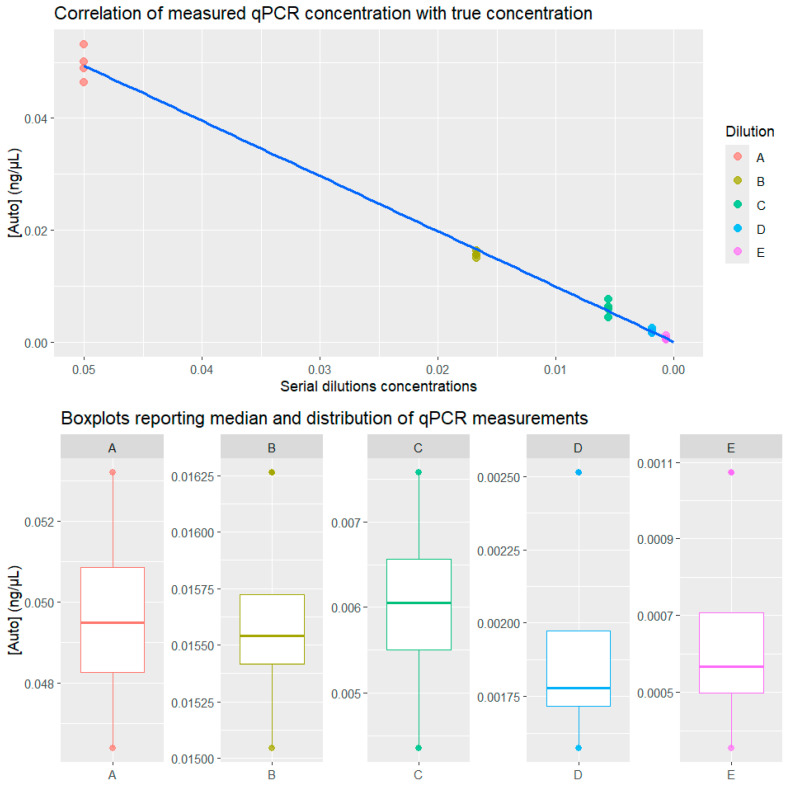
PowerQuant^®^ System results for the [Auto] target of five serial three-fold dilutions. Above is the correlation of qPCR measurements with true concentration. Below, the boxplots show the median and distribution of the measurements for each dilution.

Results and metrics of the four technical replicates performed with the PowerQuant^®^ System per each dilution are shown in Table 1 and Figure 1. Accuracy was calculated as the Mean Absolute Percentage Error (MAPE) across replicates for each dilution, while precision is reported in terms of Coefficient of Variability (CV).

In general, the correlation between measured concentration ([Auto]) and true concentration was very strong (Pearson’s r = 0.997).

The PowerQuant^®^ System results were highly accurate and precise for the more concentrated dilutions (A and B) while showing an increased measurement error and a higher degree of variability for less-concentrated dilutions. Concentration and standard deviation were, as can be expected, directly proportional, with a correlation coefficient r = 0.89. Conversely, concentration was strongly inversely proportional to both MAPE (r = −0.729) and CV (=−0.661). As shown for dilution E, the qPCR assay’s accuracy decreased to a moderate accuracy equal to 76.8% (100%—MAPE). Thus, for concentrations of 0.000617 ng/μL, the measured [Auto] was, on average, 33.2% off from the true value. Conversely, for a higher concentration of 0.05 ng/μL, the measured concentration ([Auto]) was, on average, 4% off from the true concentration. qPCR and STR-PCR were demonstrated to be similarly susceptible to stochastic effects, which are more pronounced when the DNA template concentration is lower.

#### 3.1.2. Qualitative Analysis—Correlation of True Concentration with Profile Completeness

Drop-ins are stochastic effects, which are more frequent in low-template samples [4]. The highest number of drop-in alleles was observed for dilution B, with a mean of two drop-ins across the four replicates, followed by dilution C (average of 1.75 drop-ins). Dilution A, D, and E did not present any drop-in in any of the replicates. For profile completeness calculations and further assessments on peak height, the drop-ins were removed.

Profile completeness was calculated based on the number of alleles present, Amelogenin locus included, divided by the number of the alleles present in the known reference STR genotype. In this case, the product (2800 M male gDNA) was heterozygous at every locus typed by the PowerPlex^®^ ESX-17 Fast Kit, except for locus D22S1045, where it was homozygous. Results are reported in Table 2. There was a strong correlation between true concentration and profile completeness. When considering the four replicates per dilution as independent data points, the Pearson r was equal to 0.639.

#### 3.1.3. Quantitative Analysis—Correlation of True Concentration and Peak Height in RFU

For correctly assessing summary statistics, expected alleles that had dropped out were included in the genotype and were assigned a peak height = 0 RFU.

Considering each allele call as an independent data point, a very strong correlation between true concentration and peak height was observed (r = 0.840). Differences in the peak height between dilutions are all significant (Wilcoxon–Mann–Whitney test, *p*-value < 0.01 for Bonferroni correction) except for dilutions D and E for which *p*-value = 0.346 (Supplementary Table S1). Mean height in RFU and standard deviation were calculated for each replicate of each dilution. Results are reported in Supplementary Table S2. Additionally, the absolute percentage error was calculated for each detected peak height against the mean height of the respective replicate. For absolute percentage error, no statistical difference (Wilcoxon–Mann–Whitney test, *p*-value > 0.01 for Bonferroni correction) was observed for dilutions that were one the product of the other, e.g., dilutions A and B (Supplementary Table S1). The Mean Absolute Percentage Error was calculated for each replicate, and the results are shown in Supplementary Table S2. The replicates’ mean height and standard deviation were very strongly dependent on the initial DNA template (r = 0.974 and 0.944, respectively). A strong negative correlation between true concentration and MAPE (r = −0.671) and true concentration and CV (r = −0.66) was observed.

**Figure 2:**
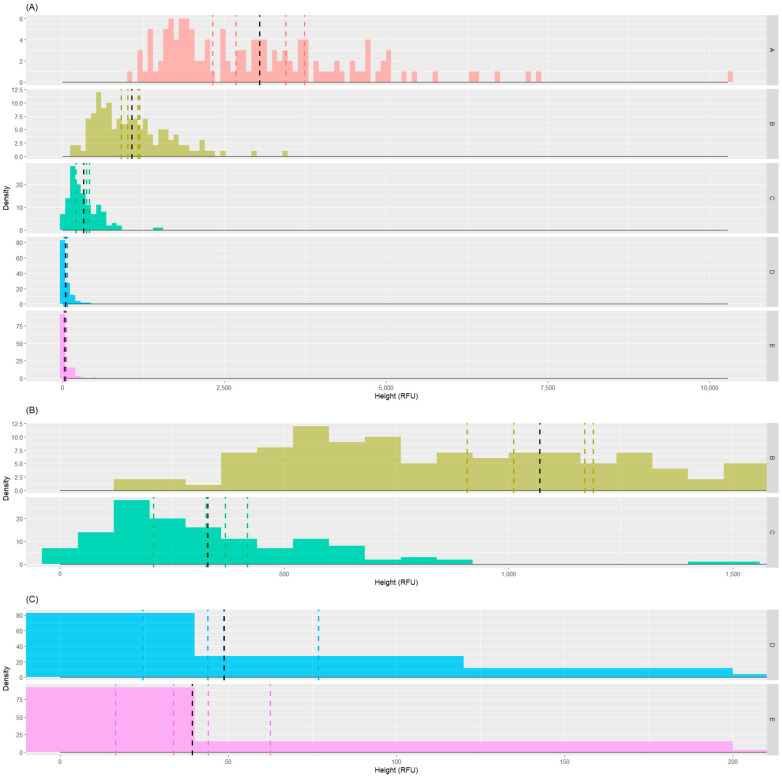
Histograms representing the distribution of peak heights across replicates for each dilution. On the y-axis, the numerosity of samples that fall within a peak height range is reported. The x-axis bin width was set to 80 RFU. The vertical black dashed line represents the overall mean peak height (RFU) calculated across all replicates, while the colored dashed lines represent the mean height of each replicate. (**A**) All dilutions; (**B**) zoomed-in view of dilutions B and C; (**C**) zoomed-in view of dilutions D and E.

All replicates were then evaluated together for each dilution. The summary statistics are reported in Table 3. In Figure 2, peak height values divided by dilutions are reported, along with the overall samples’ mean peak height and mean peak height per replicates.

The correlation values observed for the unified replicates mirror the ones obtained from the separated replicates. The correlation between true concentration and mean height appeared very strong (r = 0.9995), as well as the one with standard deviation (r = 0.9988). A strong negative correlation was observed between concentration and MAPE (r = −0.6851) and between concentration and CV (r = −0.693).

The link between profile completeness and peak height, standard deviation, and MAPE was investigated. A strong correlation was found between profile completeness and both mean peak height and standard deviation (r = 0.661 and r = 0.693, respectively). Interestingly, a very strong negative correlation existed between profile completeness and MAPE (r = −0.995).

**Figure 3:**
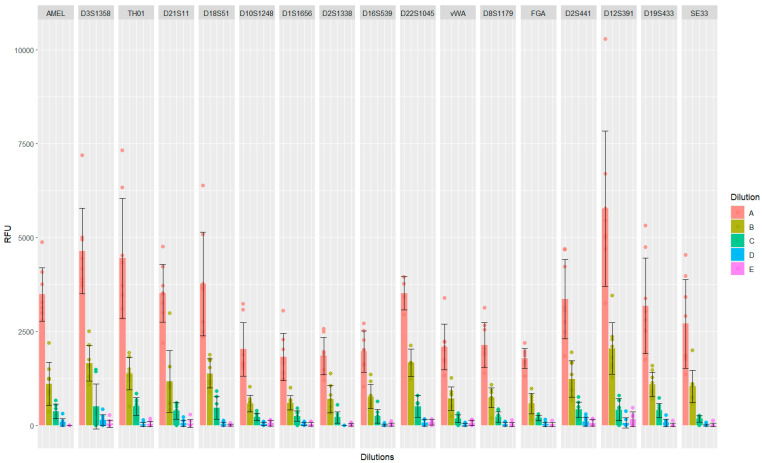
Graphical representation of the peak heights (RFU) detected per each locus across all five 2800 M dilution series samples. Each point represents a single replicate. The height of the barplot falls at the mean height (RFU) calculated for each locus across replicates, while the error bar indicates the standard deviation.

Mean peak height was calculated for each locus by grouping all replicates for each dilution (Figure 3). Similarly, peak height variation and peak height ratio (PHR) were evaluated at each locus. Results are reported in Supplementary Table S3 and are graphically represented in Supplementary Figure S1. A moderate correlation was observed between the concentration of the dilution, meaning the amount of template DNA for STR-PCR and PHR (r = 0.497). D22S1045, the only homozygous locus, should show a peak height that is around double the mean peak height observed in the other heterozygous loci. However, when compared with vWA, D8S1179, and FGA, which all shared the same fluorescence (Yellow), D22S1045 showed a peak height slightly lower than double the height of these loci. The discrepancy between expected and observed results could be due to stochastic effects of the STR-PCR reaction as well as to variation in fluorescence emission intensity for different fluorophores [38,39]. In our case, the Green (JOE dye) and Yellow (TMR-ET dye) fluorescence showed a consistently reduced field strength when compared to the Blue and Red dyes (fluorescein and CXR-ET, respectively) [31].

### 3.2. Casework Samples—Variation Assessment and Prediction of Probative Value

Casework samples showcased the expected variability in quantification results and peak height. The number of drop-out loci, mean peak height, standard deviation, MAPE, and CV were calculated for each sample (Supplementary Table S4). Mean peak height was calculated for each marker and then averaged across all loci to account for drop-outs. A general decrease in correlation strength between concentration and each metric was observed. A moderate negative correlation existed between the concentration and number of drop-out loci (r = −0.424), while a positive one existed between the concentration and mean peak height (r = 0.535).

**Figure 4:**
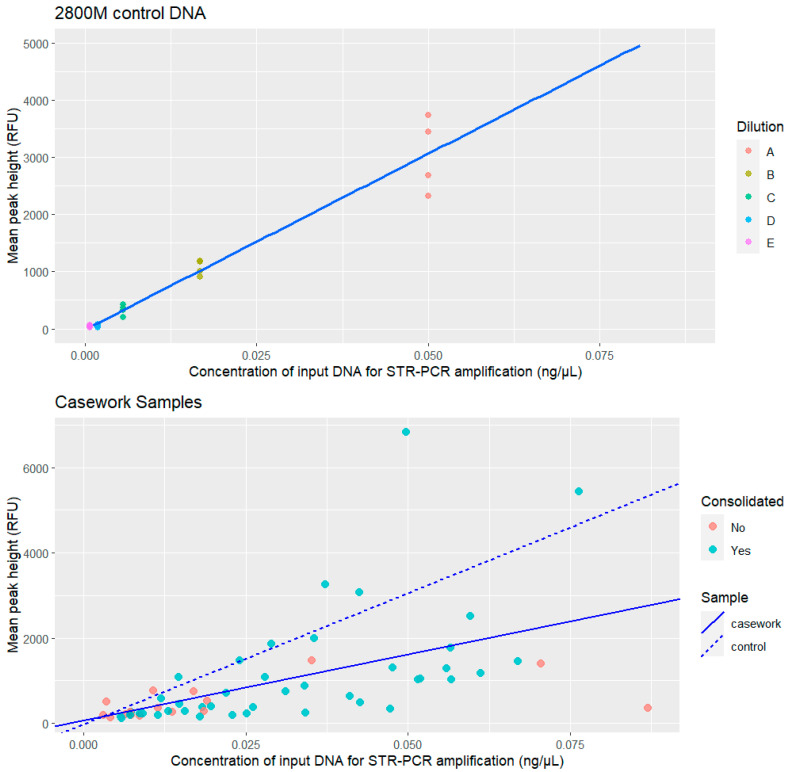
Correlation of mean peak height versus DNA template concentration for the replicates of the control 2800 M control DNA dilution series (above) and of the casework samples (below). The correlation of mean peak height for casework samples with DNA template (continuous line) is compared with the regression line fitted on the 2800 M control DNA dilution series (dashed line).

As can be observed from Figure 4, the slope of the linear correlation between mean peak height and DNA template concentration was smaller than the one calculated for the 2800M control DNA, indicating a decrease in mean peak intensity (RFU) despite a higher amount.

The samples were grouped according to whether they yielded or not a probative, consolidated profile in at least 10 loci. Student’s t-test was performed on the concentration results ([Auto]) between the two groups. A statistical difference existed in terms of qPCR results and profile completeness (*p*-value < 0.05). The casework samples dataset was split into a training set (48 samples) and a test set (12 samples). The training set was used to model a logistic regression that correlated the samples’ [Auto] concentration and their probative value (Yes = 10 or more consolidated loci, No = less than 10 consolidated loci). The model’s predictive value was tested on the test set returning a McFadden Pseudo R^2^ of 0.161 and an optimal cut-off value at 0.551, corresponding to an [Auto] = 0.001165 ng/μL. This means that if a sample, based on its concentration, had a 55% probability of being probative, it would be categorized as such. As expected, for such a low threshold, the sensitivity was 0.857, while the specificity was 0.4, along with a miscategorization error of 25%. Despite their higher-than-average concentration, some of the samples yielded no probative profile. Degradation (DI) and purity (IPC Shift) were investigated in terms of their contribution to a correct sample categorization. A statistical difference was observed for DI (*p*-value < 0.05) but not for the IPC Shift. A second model was trained using three predictors: [Auto], DI, and IPC Shift. The model (McFadden Pseudo R^2^ = 0.132) returned an optimal cut-off probability of 0.042, thus slightly decreasing sensitivity and specificity to 0.786 and 0.2, respectively. The results demonstrate how a sufficient DNA template concentration is crucial for STR typing success, albeit not the only determining factor. To gain a deeper understanding of the combined impact of DNA quantity, degradation, and contamination on the informative value of an STR profile, further research is required on a broader range of samples.

## 4. Discussion

A series of five three-fold dilutions of the 2800 M control DNA (Promega, Madison, WI, USA), falling way below the optimal DNA input for STR-PCR with the PowerPlex^®^ ESX-17 Fast kit (Promega, Madison, WI, USA), was set up. The PowerQuant^®^ System’s accuracy and precision were evaluated by quantifying the five dilutions in four replicates. Results were confirmed to be highly accurate and precise [10] for more concentrated samples while showing an increased measurement error and a higher degree of variability inversely proportional to the true concentration of the dilution. For less-concentrated samples, the increased Mean Absolute Percentage Error (MAPE) and Coefficient of Variation (CV) showed how the qPCR precision was affected by the initial DNA amount. Results indicate how the laboratory workflow may benefit from performing technical replicates of the quantitation step for a more accurate quantitative assessment of smaller quantities of DNA template. Each dilution was STR-PCR-amplified four times. Qualitative and quantitative characteristics of the respective EPGs, such as profile completeness, number of drop-ins, mean peak height (and relative descriptive metrics), and peak height ratio, were described by considering each single replicate and grouping the replicates by dilution. Results were correlated with the true concentration of the sample. Profile completeness was strongly dependent on the initial input amount due to the well-known stochastic events of STR-PCR, more prominent for scarce DNA templates [4]. At the same time, drop-in peaks were observed only for intermediate dilutions, possibly meaning that the DNA amount in more diluted samples was insufficient to produce detectable inherent PCR errors.

Quantitatively, the mean peak height (in RFU) calculated for each dilution was very strongly correlated with true concentration, confirming the linear correlation between the two measures previously reported in the literature for samples of higher concentration [12,20]. Similar to what was observed for qPCR results, the variation in peak height observed among replicates of the same sample tended to increase with decreasing DNA template amount. These results indicate that qPCR and STR-PCR are both similarly susceptible to stochastic errors, with the intensity of these errors inversely proportional to the initial sample concentration. The increased variation in peak height ratio observed at each locus for smaller DNA quantities and the strong negative correlation between profile completeness and peak height mean percentage error (r = −0.995) support the importance of performing multiple STR-PCR reactions, especially for trace DNA samples. The smaller the amount of DNA template is, the less complete the resulting STR profile will be, leading to greater peak height differences from the mean because of the presence of drop-out loci that will introduce extreme values (0 RFU).

Based on the results obtained from the control DNA dilutions, quantitative and qualitative data from 60 casework samples were analyzed. The aim was to evaluate whether the correlation observed for undegraded, ideal samples was present in real casework samples, inherently more complex, and whether it would be possible and useful to establish a quantitation threshold for discriminating probative from non-probative DNA samples. Mean peak height, MAPE, and CV calculated across STR-PCR replicates for each sample returned to be moderately correlated to [Auto] results. These results could be explained by the effect of other factors besides DNA template amount (e.g., degradation and inhibition) on obtaining a probative DNA profile. These observations were confirmed by the performance of the two logistic regression models, one based only on [Auto] as a predictor, and the other based on [Auto] and DI. Ultimately, quantitation results may not be reliable in establishing a priori a sample’s suitability for comparison. A quantitation threshold may lead to the exclusion of higher-quality probative samples, merely because of the scarce DNA. Conversely, highly degraded and contaminated samples may not yield a comparable EPG, albeit exhibiting a higher concentration of small-target fragments. From a conservative point of view, it would be better to STR type any low-concentration traces to not exclude possibly informative samples. Indeed, if a sample was typed at multiple loci, PCR replicates could be performed to confirm the results. Additionally, the usefulness of qPCR results is not limited to the pre-assessment phase for estimating the optimal DNA input for STR-PCR. Qualitative and quantitative data inferred from qPCR may be useful in interpreting and corroborating STR typing results. In fact, discrepancies may arise between the expected result and the obtained STR profile, from the reduced correlation between the amount of amplifiable DNA and the comparability of a trace existing in real casework samples. Therefore, the natural next step in this project will be the evaluation of the compound effects that degradation, inhibition, and other variables have on the informativeness of an STR profile.

## 5. Conclusions

Samples’ quantification is a crucial step in the forensic genetics laboratory’s workflow for assessing the optimal amount of template DNA for STR typing. The study demonstrated how qPCR is susceptible to the same stochastic effects and inherent reaction inefficiencies for smaller input DNA concentrations. The percentage measurement error was inversely proportional to true DNA concentration. For quantitative assessment purposes, the analysis of scarce samples may benefit from performing the qPCR step in technical replicates.

The DNA quantitation step may often offer the opportunity for sample pre-assessment and the exclusion of possibly uninformative samples from further analysis. The linear correlation between qualitative and quantitative features of an EPG with the amount of DNA template exists for undegraded samples. However, the quantitative and qualitative data collected from casework samples confirm that the amount of amplifiable nuclear DNA is not the only predictor for retrieving a probative STR profile. Degradation, contamination, and other inherent characteristics of a trace, together with STR-PCR stochastic errors, have compound effects on STR typing results, especially for lower-concentration DNA templates. Therefore, an internal threshold based on qPCR [Auto] data may be difficult to establish for correctly and consistently discriminating between probative and non-probative values. Informative samples may be excluded on the basis of a low qPCR-measured DNA concentration, while their good quality may ensure a successful typing. Conversely, samples richer in short-length fragments but of poorer quality may be PCR-amplified, resulting in profiles not suitable for comparison. From a conservative point of view, the explorative STR typing of samples of any given quality and quantity ensures that no probative data are excluded.

In conclusion, qPCR results may not be as useful for sample pre-assessment as they are for interpreting and corroborating STR typing results and for clearly illustrating them to the stakeholders. Especially for complex traces, the quantitative and qualitative data derived from a PCR-based quantification system provide information on a trace’s characteristics that better explain and contextualize the STR results in light of the inherent PCR reaction process and inefficiencies. For this reason, accurate, precise, and sensitive qPCR systems, such as the PowerQuant^®^ System (Promega) and Quantifiler™ Trio Quantification System (Thermo Fisher Scientific), should be employed, coupled with replicate analysis to account for the measurement variability observed at a greater extent in samples of poor quantity and quality.

## Figures and Tables

**Table 1:** Precision and accuracy metrics for qPCR results were calculated for each replicate in a dilution.

Dilution	True Concentration (ng/μL)	Mean [Auto] (ng/μL)	SD [Auto] (ng/μL)	MAPE	CV
A	0.05	0.049648	0.002829	4.0%	0.057
B	0.0167	0.015598	0.000502	6.0%	0.032
C	0.0055	0.006011	0.001326	19.7%	0.221
D	0.00185	0.001912	0.000413	14.6%	0.216
E	0.000617	0.00064	0.000306	33.2%	0.478

**Table 2:** Profile completeness values for each replicate of all samples in the 2800M control DNA dilution series. SD = Standard Deviation.

Dilution	Rep 1	Rep 2	Rep 3	Rep 4	Consensus Profile	Mean (Rep 1–4)	SD
A	100%	100%	100%	100%	100%	100%	0
B	100%	100%	100%	100%	100%	100%	0
C	91.2%	94.1%	94.1%	94.1%	94.1%	93.4%	1.45%
D	44.1%	38.2%	26.5%	23.5%	38.2%	33.1%	9.71%
E	17.6%	32.4%	32.4%	38.2%	32.4%	30.2%	8.8%

**Table 3:** Peak height metrics calculated on united replicates. SD = Standard Deviation; MAPE = Mean Absolute Percentage Error, CV = Coefficient of Variation. The MAPE was calculated as the average percentage error of each peak height from the mean peak height.

Dilution	Mean Height (RFU)	SD (RFU)	MAPE	CV
A	3046.66	1503.66	35.6%	0.494
B	1068.92	585.29	42.4%	0.548
C	329.79	250.61	52.1%	0.760
D	48.75	82.86	128.5%	1.7
E	39.34	76.91	145%	1.955

## Data Availability

The raw data supporting the conclusions of this article will be made available by the authors on request.

## Supplementary Materials

The following supporting information can be downloaded at: https://www.mdpi.com/article/10.3390/genes15060744/s1, Table S1: p-values calculated for individual peak height and Absolute Percentage Error for each sample in the dilution series; Table S2: Peak height metrics calculated on separated replicates; Table S3: Peak heigh metrics for each locus; Table S4: Casework samples data and metrics; Figure S1: Peak Height Ratio (PHR) at each locus for replicates grouped by dilution.
