# Supplementary material for: The Usefulness of qPCR Data for Sample Pre-Assessment and Interpretation of Genetic Typing Results
Source: Genes (Basel). 2024 Jun 5;15(6):744. doi: 10.3390/genes15060744 (PMC11203103; doi:10.3390/genes15060744)
Supplement: Supplementary file 1 [file genes-15-00744-s001.zip › genes-3037006-supplementary.pdf]

The Usefulness of qPCR Data for Sample Pre-Assessment and Interpretation of Genetic Typing Results

SUPPLEMENTARY MATERIALS

Individual peak height	A	B	C	D	E
A	1				
B	2.45E-30	1			
C	1.05E-43	1.50E-28	1		
D	1.01E-47	5.46E-42	1.67E-24	1	
E	7.41E-48	2.32E-42	1.10E-25	0.346258	1
**Absolute Percentage Error**	A	B	C	D	E
A	1				
B	5.51E-02	1			
C	5.08E-04	5.02E-02	1		
D	1.78E-19	2.87E-17	1.24E-13	1	
E	1.57E-16	5.78E-15	1.70E-12	0.250326	1

Table S 1: *p*-values calculated for individual peak height and Absolute Percentage Error for each sample in the dilution series. After establishing data non-normality, statistical significance was carried out with Wilcoxon-Mann-Whitney test.

Dilution	Replicate	True concentration (ng/μL)	Mean peak height (RFU)	SD (RFU)	MAPE	CV
A	1	0.05	2681.12	1085.88	35%	0.41
A	2	0.05	2317.61	970.68	35.3%	0.42
A	3	0.05	3739.09	1810.42	34%	0.48
A	4	0.05	3448.82	1584.24	38.6%	0.46
B	1	0.0167	1011.3	463.33	37.3%	0.46
B	2	0.0167	907.3	500.32	44.6%	0.55
B	3	0.0167	1188.06	716.81	44.2%	0.6
B	4	0.0167	1169	606.81	43.4%	0.52
C	1	0.0055	418.16	355.01	62.9%	0.85
C	2	0.0055	326.21	222.56	52.5%	0.68
C	3	0.0055	208.48	164.06	53.6%	0.79
C	4	0.0055	369	178.95	40%	0.48
D	1	0.00185	76.76	120.77	112.3%	1.57
D	2	0.00185	48.73	70.33	121.2%	1.44
D	3	0.00185	24.69	48.28	150%	1.96
D	4	0.00185	43.94	68	131.2%	1.55
E	1	0.000617	16.56	42.38	168.8%	2.56
E	2	0.000617	44.03	76.05	139.4%	1.73
E	3	0.000617	62.56	110.6	131.5%	1.77
E	4	0.000617	33.7	55.65	140%	1.65

Table S 2: Peak height metrics calculated on separated replicates. Mean peak height (RFU) was calculated for each STR-PCR replicate of all samples of the 2800 M control DNA dilution series, along with Standard Deviation (SD), Mean Absolute Percentage Error (MAPE), and Coefficient of Variation (CV). The MAPE was calculated as the average percentage error of each peak height from the mean peak height.

	A				B				C			
	Mean height (RFU)	SD (RFU)	MAPE	PHR	Mean height (RFU)	SD (RFU)	MAPE	PHR	Mean height (RFU)	SD (RFU)	MAPE	PHR
AMEL	3482.75	704.59	22.5%	98.3 %	1107.38	570.5	37.1%	70.4 %	377.38	195.79	37.6%	94.8 %
D3S1358	4640.25	1139.2	53.6%	97.4 %	1652.5	468.78	60%	75.4 %	506	604.6	99.3%	74.7 %
TH01	4449.5	1593.46	44.7%	89.5 %	1374.88	432.27	38.6%	72.8 %	501	235.64	78.2%	74.1 %
D21S11	3515.75	772.41	17%	74.3 %	1168.25	818.38	46.1%	70.2 %	388.75	218.06	60.8%	54.3 %
D18S51	3760.12	1378.79	27%	97.1 %	1382	381.6	35.1%	60.7 %	467.71	300.45	60.1%	59.5 %
D10S1248	2023.25	703.84	33.9%	92.2 %	585.88	217.35	46.2%	75.9 %	224.88	101.17	35.2%	82.5 %
D1S1656	1820.88	619.1	41.2%	96.3 %	602.38	187.1	42.2%	69.1 %	248.75	134.3	37.1%	79.6 %
D2S1338	1847.62	499.31	39%	100 %	700.62	363.36	40.8%	100 %	215.38	147.56	44.3%	100 %
D16S539	1966.25	542.61	36%	97.8 %	771.25	320.18	32.6%	85.3 %	243.88	177.86	41.2%	75.8 %
D22S1045	3513.5	444.03	31.3%	82.9 %	1664	364.99	55.8%	95.7 %	509.5	296.62	100%	45.1 %
vWA	2083.62	604.29	29.4%	78.7 %	717.25	309.47	37.2%	89.4 %	182.75	104.65	42.3%	70.5 %
D8S1179	2139.25	588.14	29.8%	90.7 %	742.88	262.53	33.2%	77.2 %	225.75	137.02	39.8%	76.6 %
FGA	1779.5	261.05	39.8%	99.1 %	582	274.4	46.8%	64.7 %	201.12	68.97	35.2%	78.8 %
D2S441	3353.38	1056.54	18.1%	86.8 %	1237.75	481.01	39.5%	86.3 %	422.25	202.08	41.6%	94.7 %
D12S391	5769.62	2071.24	87.7%	96.2 %	2048.62	680.52	89.6%	81.3 %	420.38	291.6	84.5%	74.9 %
D19S433	3180.25	1267.65	28.3%	95.7 %	1088.88	327.93	20.8%	81.6 %	401.25	176.76	30.8%	96.2 %
SE33	2701.12	1179.13	23.8%	79.1 %	1042.62	433.2	25.6%	88.7 %	176.88	82.88	43%	30 %

	D					E				
	Mean height (RFU)	SD (RFU)	MAPE	PHR	Comment	Mean height (RFU)	SD (RFU)	MAPE	PHR	Comment
AMEL	82.38	102.17	134.6%	20.9 %		0	0	100%	NA	Locus d.o.
D3S1358	140.88	143.59	245.5%	85.8 %		47.62	97.89	145.1%	49.7 %	
TH01	28.12	53.57	99.4%	46.8 %		36.62	70.87	184.1%	0 %	Allele d.o.
D21S11	58.12	86.23	148%	NA	Locus d.o.	50.12	102.8	140.1%	100 %	
D18S51	37.25	54.3	106.1%	30.1 %		8.12	22.98	99.1%	0 %	Allele d.o.
D10S1248	39.88	43.63	93%	79.5 %		57.12	67.08	110.6%	0 %	Allele d.o.
D1S1656	36.38	40.59	68.9%	87.6 %		25.38	47.02	116.8%	0 %	Allele d.o.
D2S1338	0	0	100%	100 %		17.5	32.4	116.8%	100 %	
D16S539	8.62	24.4	88.8%	31 %		21.38	41.86	104.2%	0 %	Allele d.o.
D22S1045	76	88.3	245.7%	0 %	Allele d.o.	80.75	67.5	221.9%	0 %	Allele d.o.
vWA	15.75	29.48	84.2%	0 %	Allele d.o.	65.5	70.89	201.8%	0 %	Allele d.o.
D8S1179	27.86	47.72	93.6%	0 %	Allele d.o.	25.25	50.16	107.3%	0 %	Allele d.o.
FGA	32.71	61.26	149.6%	59.3 %		20.33	49.81	99.2%	63.2 %	
D2S441	104.12	120	189.9%	77.5 %		68.86	86.33	237.3%	97.9 %	
D12S391	67.38	138.26	157%	0 %	Allele d.o.	162.67	196.1	316.5%	58.4 %	
D19S433	73.38	96.09	148.7%	84.5 %		15.25	43.13	120.2%	43.8 %	
SE33	8.88	25.1	88.4%	0 %	Allele d.o.	16.75	47.38	124.7%	78.9 %	

Table S 3: Peak height metrics for each locus. Mean peak height, standard deviation (SD), Mean Absolute Percentage Error (MAPE), and Peak Height Ratio (PHR) at each locus for each sample in the 2800 M control DNA dilution series

Sample	[Auto] (ng/μL)	DI	IPC Shift	drop-out loci	Consolidated	Mean height (RFU)	SD (RFU)	MAPE	CV
Case.001	0.067	5.65	0.28	0	Yes	1455.41	1329.31	72.1%	1.2
Case.002	0.012	5.86	0.18	2	Yes	586.87	887.28	79.5%	1.3
Case.003	0.028	3.28	-0.14	0	Yes	1078.6	875.9	64.5%	0.8
Case.004	0.0115	NA	-0.27	12	No	366.36	309.34	66.4%	0.9
Case.005	0.0183	6.84	-0.19	1	Yes	381.715	500.97	81%	1.3
Case.006	0.0072	NA	-0.15	12	No	257.12	278.62	69.1%	1
Case.007	0.0186	34.07	0.13	8	No	283.595	273.01	69.55%	1
Case.008	0.0197	6.7	-0.17	3	Yes	391.96	398.01	77.95%	1.25
Case.009	0.0065	5.46	-0.24	11	No	217.73	211.89	71%	1.05
Case.010	0.0087	NA	0.39	16	No	170.71	68.41	44.6%	0.5
Case.011	0.0515	2.92	-0.33	0	Yes	1031.31	905.88	76.3%	1.1
Case.012	0.0091	7.31	-0.09	5	Yes	230.57	150.885	57.1%	0.8
Case.013	0.0073	NA	-0.04	17	No	190.08	107.89	50.7%	0.6
Case.014	0.0137	NA	0.06	15	No	256.31	138.97	46.9%	0.5
Case.015	0.0004	NA	2.58	NA	No	NA	NA	NA	NA
Case.016	0.0107	20.34	-0.03	8.5	No	772.095	1087.375	87.4%	1.5
Case.017	0.017	31.9	-0.05	5	No	757.61	1272.64	89.8%	1.5
Case.018	0.0035	14.21	-0.13	7	No	503.835	573.74	75.15%	1.1
Case.019	0.0566	16.63	-0.24	0	Yes	1771.17	2792.42	93.3%	1.5
Case.020	0.029	9.7	-0.13	2	Yes	1870.71	2939.165	102.9%	1.6
Case.021	0.0411	8.13	0.52	0	Yes	640.96	1098.98	87.7%	1.5
Case.022	0.0476	NA	0.73	0	Yes	1304.64	1544.6	87.7%	1.5
Case.023	0.0241	NA	0.39	0	Yes	1474.99	1538.85	89.6%	1.4
Case.024	0.0312	6.89	0.5	0	Yes	754.82	1064.83	78.4%	1.2
Case.025	0.0087	3.79	0.22	1	Yes	230.66	247.69	49.8%	0.9
Case.026	0.0007	NA	1.56	NA	No	NA	NA	NA	NA
Case.027	0.0148	3.14	0.73	0	Yes	444.14	240.47	51.4%	0.7
Case.028	0.003	3.9	-0.16	8	No	185	109.03	43.5%	0.6
Case.029	0.0425	1.84	-0.08	0	Yes	3077.845	2479.025	58.45%	0.85
Case.030	0.0372	1.48	-0.04	0	Yes	3267.43	1999.56	41.5%	0.6
Case.031	0.0073	4.15	0.2	2	Yes	262.07	163.95	57.9%	0.7
Case.032	0.0356	3.55	0.33	1	Yes	1989.85	1948.19	96.1%	1.4
Case.033	0.0705	1.05	0.31	9	No	1390.38	1215.01	93.6%	1.3
Case.034	0.0352	NA	0.19	7	No	1465.15	1686.21	82.9%	1.2
Case.035	0.0764	2.92	-0.2	0	Yes	5440.2	3721.68	70.4%	1
Case.036	0.087	5.55	0.35	3	No	357.255	276.75	67.95%	0.9
Case.037	0.0191	11.32	0.45	4	No	522.94	542.84	81.5%	1.3
Case.038	0.0341	3.9	0.04	0	Yes	884.88	881.98	82%	1.2
Case.039	0.056	4.06	0.29	0	Yes	1291.02	1422.74	82.6%	1.2
Case.040	0.0115	3.94	-0.02	0	Yes	182.29	106.5	55.9%	0.7
Case.041	0.0473	3.66	-0.05	0	Yes	347.98	237.42	75.3%	1
Case.042	0.0612	4.78	-0.08	0	Yes	1174.98	1611.51	91.1%	1.4
Case.043	0.0342	4.38	0.08	0	Yes	239.37	142.18	63.1%	0.9
Case.044	0.0229	11.59	0.49	1	Yes	190.83	154.21	67.1%	1
Case.045	0.0497	5.3	0.06	0	Yes	6832.99	6842.91	77.7%	1
Case.046	0.0596	3.17	0.15	0	Yes	2520.85	3657.3	95.3%	1.5
Case.047	0.0072	8.31	-0.01	3	No	195.6	180.39	61%	0.9

Case.048	0.022	4.91	0.3	0	Yes	709.55	654.71	71.8%	1
Case.049	0.0041	8.35	0.14	9	No	130.9	90.45	45.2%	0.7
Case.050	0.0426	8.03	0.21	0	Yes	480.15	449.98	82.7%	1
Case.051	0.0519	4.62	0.37	0	Yes	1043.57	947.68	87.7%	1.2
Case.052	0.0147	9.34	0.21	2	Yes	1076.48	932.13	64.4%	0.9
Case.053	0.013	5.34	-0.1	1	Yes	284.47	198.26	64.5%	0.8
Case.054	0.0058	3.91	0.3	0	Yes	124.56	73.86	50.3%	0.7
Case.055	0.0251	3.06	-0.04	1	Yes	234.87	228.54	59.3%	0.8
Case.056	0.0179	2.88	0.12	0	Yes	159.8	113.65	54.6%	0.9
Case.057	0.0261	3.47	0.07	0	Yes	384.68	321.43	76.4%	1.2
Case.058	0.0567	4.71	-0.09	0	Yes	1035.93	898.19	73.1%	1
Case.059	0.0057	3.75	0.3	3	No	147.29	86.28	60.3%	0.9
Case.060	0.0156	2.03	0.41	0	Yes	276.23	168.09	63.7%	0.9

Table S 4: Casework samples data and metrics. Quantitation [Auto] results, Degradation Index (DI), presence of PCR inhibitors (IPC Shift), number of drop-out loci, information on the probative value of the trace and peak height metrics are reported.

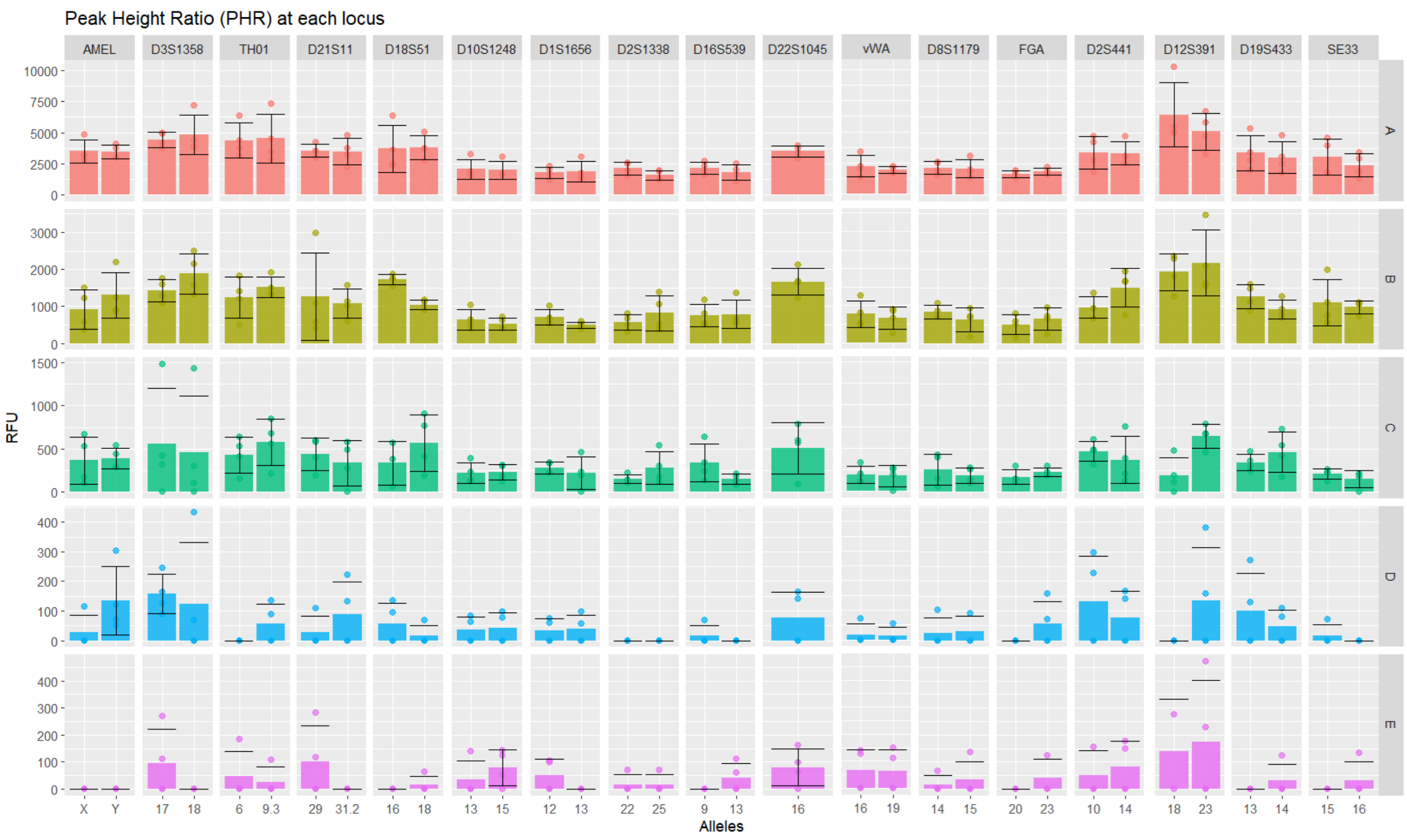

Figure S 1: Peak Height Ratio (PHR) at each locus for replicates grouped by dilution.
